# Unveiling of phosphodiesterase-5 hot residues binding to xanthine derivatives for erectile dysfunction therapy: A computational drug repurposing approach

**DOI:** 10.1371/journal.pone.0336267

**Published:** 2025-11-13

**Authors:** Amin O. Elzupir, Sondos A. J. Almahmoud

**Affiliations:** Chemistry Department, College of Science, Imam Mohammad Ibn Saud Islamic University (IMSIU), Riyadh, Kingdom of Saudi Arabia; The Islamia University of Bahawalpur Pakistan, PAKISTAN

## Abstract

Overexpression of phosphodiesterase 5 (PDE-5) presents a compelling target for the therapy of erectile dysfunction. Sildenafil and other conventional PDE-5 inhibitors may lead to adverse effects, including visual disturbances and migraines. Therefore, the investigation of novel inhibitors with enhanced safety profiles is imperative. This research employed a computational drug repurposing approach to assess US-FDA-approved xanthine derivatives (XDs) for their efficacy in targeting PDE-5. XDs exhibit a favorable affinity for the active site of the PDE-5 receptor, with binding scores between −10.0 kcal/mol and −6.3 kcal/mol for linagliptin and theobromine, respectively. The top-ranked docked Xds then underwent 300-nanosecond molecular dynamics simulations. Linagliptin demonstrated greater stability in the binding pocket (RMSD = 1.60 ± 0.34) compared to the typical inhibitor sildenafil (RMSD = 1.70 ± 0.27). The findings were corroborated by MM-PBSA calculation, which showed that linagliptin’s binding free energy of −45.6 ± 4.3 kcal/mol comparable with sildenafil’s −49.0 ± 3.1 kcal/mol. This value is notably higher than that of the deprotonated form of sildenafil, which is present at a 37.06% ratio at physiological pH 7.4. Additionally, we used per-residue energy decomposition to identify crucial residues for PDE-5 activity and thoroughly investigated hydrogen bond occupancy. This study points outthe potential of linagliptin as a PDE-5 inhibitor, paving the way for the development of a safe treatment for erectile dysfunction.

## 1. Introduction

The degradation of the intracellular second messengers cyclic guanosine monophosphate and cyclic adenosine monophosphate is one of the numerous physiological functions performed by the eleven nucleotide families that constitute the superfamily referred to as phosphodiesterases (PDEs) [1,2]. PDEs serve as potential therapeutic targets for a variety of conditions, such as heart failure, cardiac hypertrophy, depression, asthma, inflammation [1,3,4]. They are also pivotal in the treatment of erectile dysfunction (ED) and pulmonary hypertension [5–7].

Among these, phosphodiesterase 5 PDE-5 is present in various human tissues, such as muscles, lungs, brain, heart, kidney, urethra, and penis [8–13]. PDE-5 regulates cellular levels of cyclic guanosine monophosphate, a crucial signaling molecule involved in various physiological processes [1,11]. Dysregulation of cGMP leads to a decrease in intracellular calcium levels, which in turn causes the relaxation of vascular smooth muscle [1]. Consequently, PDE-5 inhibitors have become the cornerstone for treating ED. Beyond ED, PDE-5 inhibitors are also administered to patients with pulmonary hypertension due to their ability to lower blood pressure and increase blood flow to the lungs. Furthermore, accumulating evidence suggests that PDE-5 inhibitors may offer therapeutic benefits for several cancer types, including papillary thyroid, lung, prostate, esophageal adenocarcinoma, and cell carcinoma [14–16]. They also show promise in conditions like glioblastoma multiforme, Alzheimer-like pathology, heart disease, diabetes, and cognitive disorders such as dementia [6,17,18].

In 1998, sildenafil, marketed as Viagra, became the first oral PDE-5 inhibitor prescribed for ED. Tadalafil, vardenafil, and avanafil subsequently followed as approved PDE-5 inhibitors [10,12,13]. Nonetheless, current PDE-5 inhibitors exhibit specific physiological adverse effects, including visual disturbances and headaches, primarily attributed to non-specific interactions with PDE-6 or PDE-11 [10,12,13]. Consequently, identifying new PDE-5 inhibitors with an improved safety profile is paramount.

**Scheme 1:**
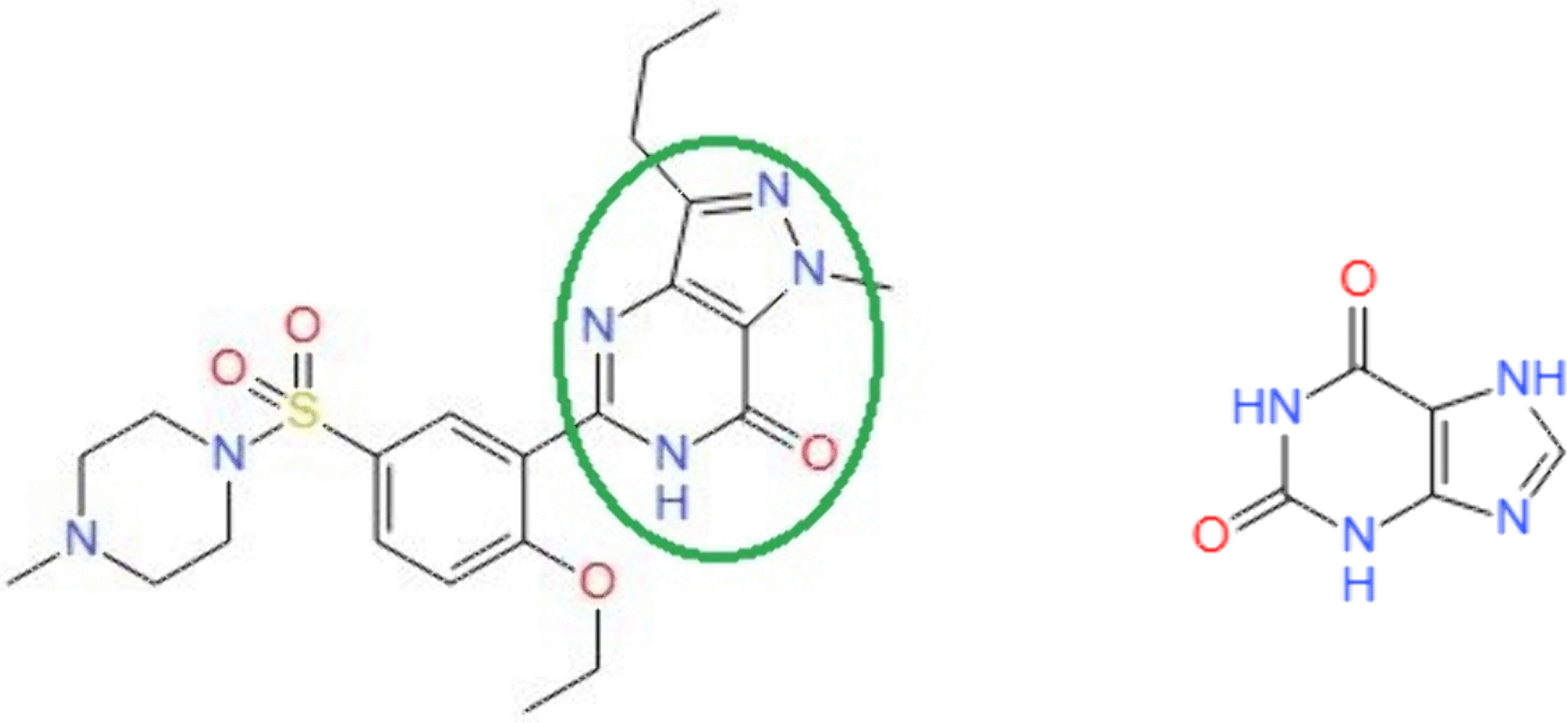
Sildenafil and xanthine chemicals structure.

Sildenafil possesses a xanthine-like moiety that contributes to its chemical structures. Consequently, medicines containing xanthine may have bioactivity comparable to that of sildenafil (Scheme 1). On the other hand, traditional medicine has long made use of xanthines, which are all-natural compounds [19]. Their high hydrophilicity facilitates rapid elimination from the body, a feature improves its potential as a favored binding substance for the zinc and magnesium minerals present in the active pocket of PDE-5, along with particular residues that increase its affinity for polar molecules [20]. This study investigates the potential use of xanthine derivatives as PDE-5 inhibitors utilizing computational drug repurposing approach.

## 2. Materials and methods

### 2.1. Building up target ligands

The mol2 files of the investigated ligands have been built up in UCSF Chimera. The process involved retrieving the SMILES strings of xanthines and the reference inhibitor sildenafil from the PubChem database online. The Molecular Modeling Toolkit has been used to minimize the energy of ligand conformation. The steepest descent and conjugate gradient are configured to 16000 steps at 0.02 Å. Subsequently, the AutoDock Tools from the Molecular Graphics Laboratory (MGL) transformed the ligands into pdbqt files after adding Gasteiger charge [19–25].

The protonation states of the ligands under investigation were determined at a physiological pH of 7.4 to ensure accurate representation. The accomplishment was achieved through the application of the Henderson-Hasselbalch equation, utilizing experimentally reported pKa values sourced from DrugBank.

**Fig 1:**
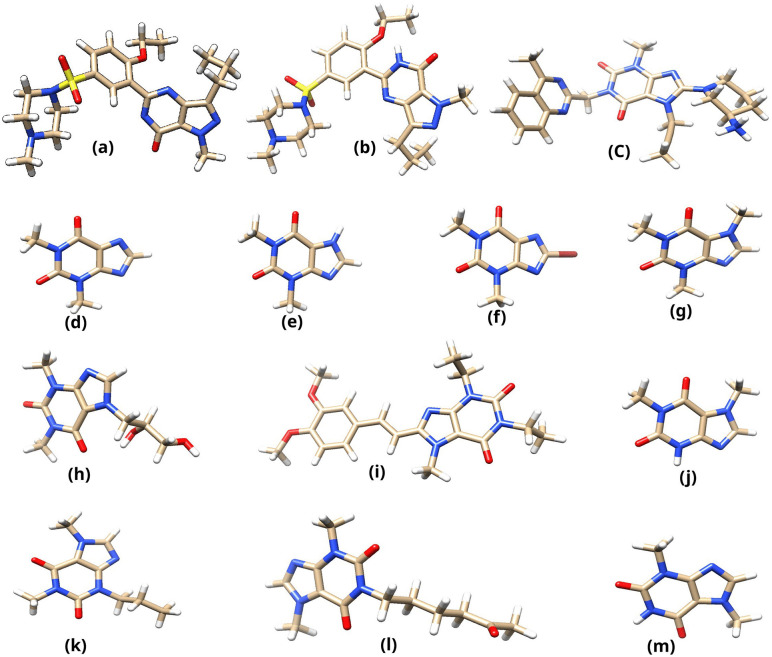
3D structures of prepared ligands: (a) deprotonated sildenafil, (b) neutral sildenafil, (c) protonated linagliptin, (d) deprotonated theophylline, (e) neutral theophylline, (f) deprotonated bromotheophylline, (g) caffeine, (h) dyphylline, (i) istradefylline, (j) paraxanthine, (k) paraxanthine N-propyl, (l) pentoxifylline, and (m) theobromine.

According to the calculations, linagliptin exists predominantly in its protonated (NH3+) form, accounting for 99.65% of its state.Theophylline and bromotheophylline were identified in their deprotonated forms at the acidic imidazole groups, with respective ratios of 27.55% and 98.51%. The elevated deprotonation rate of bromotheophylline can be attributed to the inductive effect of the bromine substituent. Sildenafil exhibits a more intricate scenario involving two ionizable sites. The acidic pyrimidone NH group contributes to a deprotonated population of 37.06%, whereas the basic site accounts for a protonated population of 3.66%. Thus, the neutral and deprotonated states of sildenafil have been investigated. The remaining xanthine derivatives were found to be predominantly neutral at pH 7.4 and were modeled as such. Fig 1 presents the prepared structures for all ligands evaluated in this study.

### 2.2. Molecular docking

The crystal form complex of sildenafil and PDE-5 was retrieved from the Protein Data Bank website (PDB ID: 2h42). Sildenafil and water molecules were eliminated by means of Autodock Tools. Then the polar hydrogens and Kollman charge were added to the PDE-5 receptor and saved in a pdbqt file format. Molecular docking was performed with MGL AutoDock Vina, with an energy range and exhaustiveness parameters of 4 and 100, respectively. The grid box size was set to 52.0, 42.0, and 36.0 Å in the 3D of the receptor, centered at coordinates 27.799, 122.752, and 9.715 Å. The [26,27]. UCSF Chimera and Discovery Studio Visualizer have been utilized for image processing and bond interactions.

### 2.3. Preparation of coordinate and topological files

The preparation of coordinate and topological files involved several steps. First, the docked ligands were separated from PDE-5 receptor using the Vina Split tool. Hydrogen atoms were then added to the ligands and saved as PDB files using MGL Autodock Tools. The inhibitors were charged and parameterized with Antechamber and Parmchk2, yielding prep and frcmod files for subsequent processing by tleap. The GAFF2 and ff14SB Amber force fields were applied to the inhibitors and the PDE-5 receptor, respectively [28,29]. System solvation and neutralization were achieved by incorporating TIP3P water molecules and sodium chloride [30]. The final coordinate and topological files for the complexes were generated using the LEaP program, a component of AmberTools 22. Before starting the simulations, a hydrogen mass repartitioning (HMR) was applied to the topology files using Parmed, in order to allows the use of a 4-fs time step without compromising the accuracy of the trajectory [31].

### 2.4. Molecular dynamics simulations

MD simulations were performed using pmemd.cuda plugin Amber 24 software [32]. The steepest descent algorithms were employed to minimize the systems for 25000 steps, followed by 25,000 steps of the more efficient conjugate gradient algorithm to achieve a refined energy minimum. Then the system was gradually heated from 0 K to 310 K over a 100-ps period within the canonical (NVT) ensemble using a Langevin thermostat. Following the heating phase, the system was equilibrated for 10 ns in the isothermal-isobaric (NPT) ensemble at a constant temperature of 310 K and a pressure of 1 bar. The pressure was maintained using a Monte Carlo barostat [33]. Finally, a 300-ns production MD simulation was conducted at 310 K with a 4-fs time step to collect trajectory data for analysis. All simulations were performed with periodic boundary conditions and the Particle Mesh Ewald (PME) method to handle long-range electrostatic interactions [34,35]. The trajectories were then analyzed using the VMD 1.9.4 and CPPTRAJ programs to calculate properties such as root-mean-square deviation (RMSD) and root-mean-square fluctuation (RMSF) [31,36,37].

### 2.5. Binding free energy calculation and decomposition analysis

To quantitatively assess the binding affinity of the ligands, the Molecular Mechanics/Poisson-Boltzmann Surface Area (MM/PBSA) and Molecular Mechanics/Generalized Born Surface Area (MM/GBSA) methods were employed using the gmx_MMPBSA package [37]. The production trajectory was processed with cpptraj. This step involved stripping all water molecules and ions, correcting for periodic boundary conditions, and saved in the GROMACS format. Concurrently, the AMBER topology of the stripped complex was converted into GROMACS format using parmed. The coordinate information provided by saving the first frame in PDB format, which used to generate index file using gmx make_ndx. The MM/GBSA analysis were conducted at the implicit solvent model, while the MM/PBSA was performed using a physiological salt concentration of 0.15 M. To provide a more complete energetic profile, the Interaction Entropy (IE) method was applied. Finally, a per-residue energy decomposition analysis was conducted to identify the specific amino acid residues that provide the most significant energetic contributions to the binding with the ligands. The binding interaction’s free energy between inhibitors and PDE-5 receptor can be derived using the subsequent equations:


ΔG=ΔH+TΔS (1)

$$\Delta \text{H}=\Delta {\text{G}}_{\text{gas}}+\Delta {\text{G}}_{\text{Sol}}$$ (2)

$$\Delta {\text{G}}_{\text{gas}}={\text{E}}_{\text{vdw}}+{\text{E}}_{\text{elec}}$$ (3)

$$\Delta \text{Gsol}={\text{E}}_{\text{pb}/\text{gb}}+{\text{E}}_{\text{np}}$$ (4)

where ΔH denotes the change in enthalpy, TΔS signifies the contribution from entropy, E_vdw_ refers to the energy associated with van der Waals interactions, E_ele_ indicates the energy from electrostatic interactions, ΔG_sol_ represents the energy related to polar solvation, and Enp pertains to the energy concerning nonpolar solvation.

## 3. Results and discussion

### 3.1 Molecular docking

**Fig 2:**
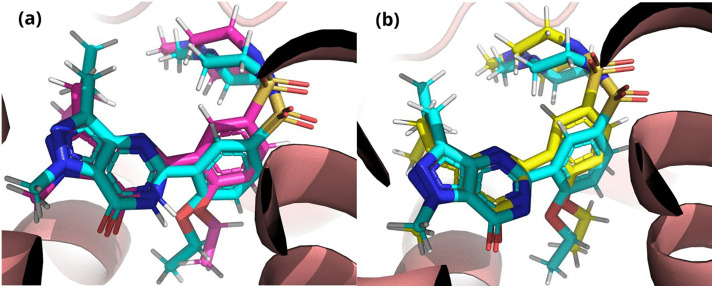
Superposition of the crystal structure and docked poses of sildenafil forms within the PDE-5 active site. The crystal ligand is depicted with its carbon skeleton in cyan. The pink docked conformer of the protonated form exhibits an RMSD of 0.45 Å, whereas the yellow docked conformer of the deprotonated form presents an RMSD of 0.47 Å.

PDE5 is a multidomain protein composed of a preserved C-terminal metal-binding catalytic site and an N-terminal domain. These domains regulate the catalytic activity and dimerization of protein [6,12]. Both the neutral and deprotonated forms of sildenafil were redocked to the phosphodiesterase-5 (PDE-5) active site to assess the reliability of the docking protocol. Fig 2 illustrates the superposition of the docked and crystal conformations of sildenafil within the PDE-5 active site. The root-mean-square deviation (RMSD) for neutral sildenafil from the crystal pose was measured at 0.46 ± 0.02 Å, accompanied by a docking score of −9.7 ± 0.0 kcal/mol. The RMSD of the protonated form was 0.5 ± 0.01 Å, accompanied by a docking score of −9.8 ± 0.1 kcal/mol. The results collectively demonstrate the suitability and reproducibility of the docking protocol.

**Fig 3:**
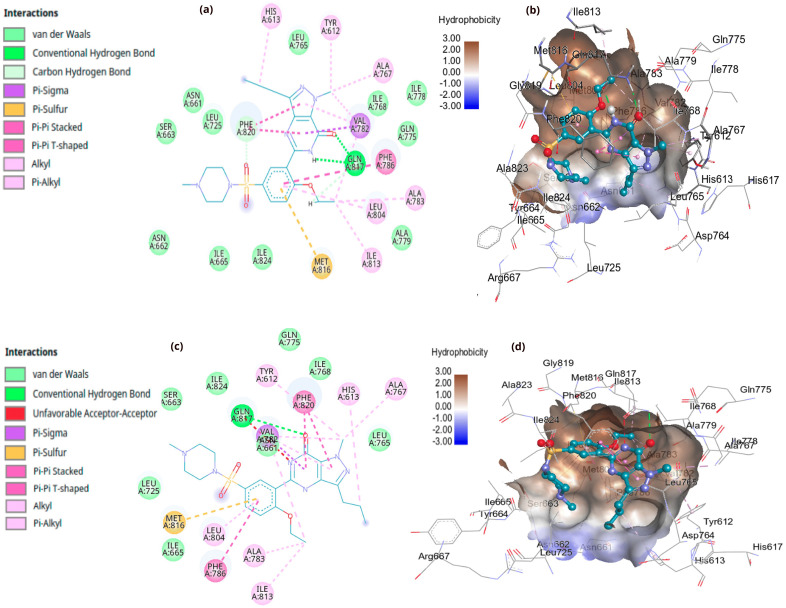
Interactions of neutral and deprotonated sildenafil within the PDE-5 active site. Panels (a) and (b) depict the neutral form in 2D and 3D, respectively. Panels (c) and (d) show the corresponding views for the deprotonated form.

It is noteworthy that, although the deprotonated form exhibits a slightly higher binding score than the corresponding neutral form, the latter demonstrates better hydrogen bonding capability to Gln 817. In contrast, the deprotonated form shows unfavorable acceptor-acceptor interactions, as illustrated in Fig 3. The hydrophobic surface surrounding sildenafil situates its position between the hydrophobic region (Met 816, Lue 804, Phe 820, etc.) and the hydrophilic region (His 613, Glu 672), characterized by various types of interactions. Both forms show Pi-Pi Stacked interactions with Phe 820 and Pi-Sulfur interactions with Met 816 and stabilized by numerous van der Waals forces (Fig 3).

**Fig 4:**
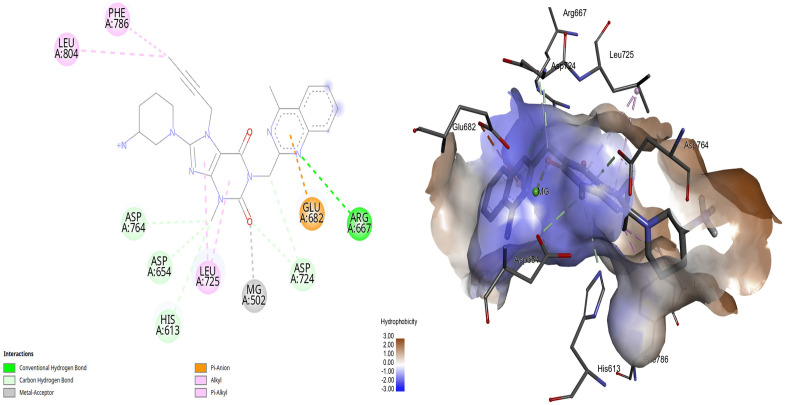
Interactions of protonated linagliptin within the PDE-5 active site in 2D and 3D.

Table 1 shows a comprehensive molecular interaction of the xanthine derivatives with the active site of the PDE-5 receptor. Linagliptin shows the best score binding energy among the investigated candidates, which is higher than that of sildenafil. This is followed by istradefylline, pentoxifylline, and dyphylline, which shown hydrogen bonds and van der Waals interactions with the hydrophobic and hydrophilic regions of the PDE-5 receptor. Notably, these hits showed a tendency to overlap with the catalytic metals of magnesium and zinc (Fig 4). In general, all xanthines and their derivatives reveal a high tendency toward the hydrophilic active pocket of the PDE-5 receptor. These include the residues of TYR 612, HIS 613, and MG 502.

**Table 1:** Binding affinity and interaction analysis of xanthine derivatives docked to phosphodiesterase 5.

Xanthine ID	Score energy kcal/mol	Hydrogen Bonds	van der Waal overlaps
Sildenafil	−9.7 ± 0.00	GLN 817	TYR 612, HIS 613, ILE 665, ASN 662, SER 663, ASN 661, LEU 725, ALA 767, VAL 782, PHE 786, ILE 824, LEU 804, ILE 813, GLN 817, PHE 820
Deprotonated Sildenafil	−9.77 ± 0.06	–	TYR 612, ASN 661, LEU 725, ALA 767, PHE 786, LEU 804, ILE 813, GLN 817, PHE 820
Protonated Linagliptin	−10.0 ± 0.00	ARG 667	MG 502, TYR 612, HIS 613, ASP 654, HIS 657, VAL 660, ASN 661, ASN 662, SER 663, ARG 667, MET 681, GLU 682, THR 723, ASP 724, LEU 725, ASP 764, LEU 765, VAL 782, PHE 786, LEU 804, MET 805, ASP 724, PHE 820
Deprotonated Bromotheophylline	−6.9 ± 0.00	ASN 662	HIS 613, HIS 657, VAL 660, ASN 661, ASN 662, MET 681, GLU 682, LEU 725
Deprotonated Theophylline	−6.53 ± 0.06	ASN 662	HIS 613, VAL 660, ASN 661, ASN 662, ASP 654, HIS 657, MET 681, GLU 682, HIS 685
Theophylline	−6.4 ± 0.00	HIS 657	HIS 613, HIS 657, VAL 660, ASN 661, ASN 662, MET 681, GLU 682
Caffeine	−6.4 ± 0.00	ASN 662	HIS 613, HIS 657, VAL 660, ASN 661, ASN 662, MET 681, THR 723, LEU 725, ASP 764
Theobromine	−6.3 ± 0.00	–	MG 502, HIS 613, HIS 657, ASN 661, ASN 662, MET 681, GLU 682
Dyphilline	−6.67 ± 0.06	GLU 682	HIS 613, VAL 660, ASN 661, ASN 662, HIS 657, MET 681, GLU 682, HIS 685
Paraxanthine	−6.4 ± 0.00	–	HIS 613, HIS 657, ASN 661, ASN 662, MET 681, GLU 682, HIS 685
Paraxanthine, N-propyl Derivative	−6.5 ± 0.00	ASN 662	MG 502, HIS 613, HIS 657, VAL 660, ASN 661, ASN 662, MET 681, LEU 725
Istradefylline	−8.9 ± 0.00	–	TYR 612, HIS 657, ARG 667, GLU 682, LEU 725, ASP 764, LEU 765, ALA 767, ILE 768, GLN 775, ILE 778, ALA 779, VAL 782, PHE 786, GLN 817, PHE 820
Pentoxifylline	−6.97 ± 0.06	–	TYR 612, HIS 613, LEU 725, LEU 765, ALA 767, ILE 768, GLN 775, VAL 782, GLN 817, PHE 820

### 3.2. Molecular dynamic simulation

#### 3.2.1 Structural stability and compactness.

**Fig 5:**
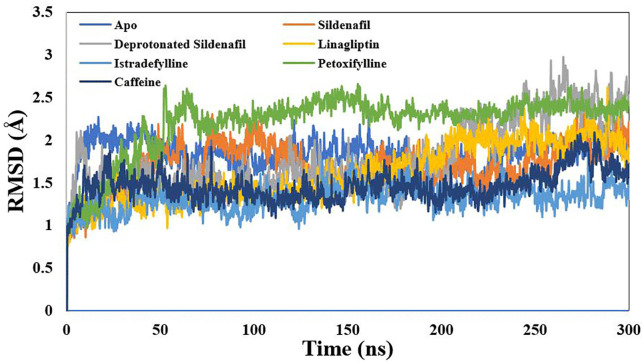
Root-mean-square deviation (RMSD) for the backbone carbon atoms of the simulated xanthine derivatives-phosphodiesterase 5 complexes based on the starting frame as a reference.

RMSDs were computed for backbone atoms with respect to the initial coordinate as a reference. The mean RMSD values of the PDE-5 complexes for the last 100 ns of the simulation are presented in Table 1, using a block-averaging approach. Sildenafil and xanthine derivatives reached the equilibration states of PDE-5 at approximately 60 ns and maintained lower RMSDs along the production runs, confirming their stability (Fig 5). Istradefylline emerged as the most effective stabilizing agent, yielding the lowest mean RMSD of 1.31 ± 0.15 Å. In contrast, pentoxifylline showed the highest mean RMSD of 2.20 ± 0.35 Å, even though it showed a stable plateau after 50 ns. Interestingly, the deprotonated form of Sildenafil showed a higher mean RMSD with a larger standard deviation of 0.41 Å, implying greater structural fluctuation and a less stable binding mode. This was not unexpected due to an unfavorable acceptor-acceptor interaction, as shown in the docking section.

**Fig 6:**
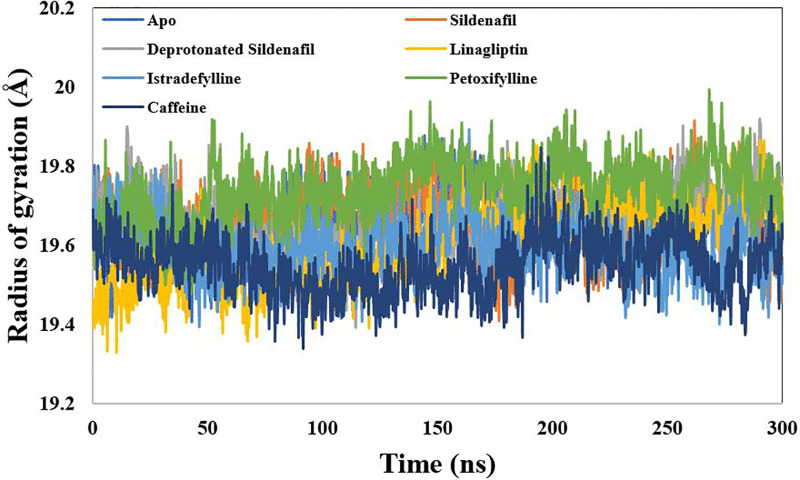
Radius of Gyration (Rg) for the simulated Xanthine Derivatives-Phosphodiesterase 5 Complexes. The compound title is located at the top.

The overall compactness of the protein structure was measured using the Radius of Gyration (Rg). As shown in Table 2, the mean Rg values are tightly clustered within a narrow range, from 19.55 Å for the caffeine-bound PDE-5 to 19.74 Å for the pentoxifylline-bound PDE-5. This consistency indicates that the protein remains stably packed and compact, regardless of whether it is in the apo state or bound to an inhibitor (Fig 6).

**Table 2:** RMSD. Rg and RMSF values for the simulated xanthine derivative-phosphodiesterase 5 complexes during 300 ns production runs.

PDE-1 complex ID	Statistic	Apo	Sildenafil	Deprotonated Sildenafil	Linagliptin	Istradefylline	Petoxifylline	Caffeine
RMSD*	mean	1.83	1.70	1.79	1.60	1.31	2.20	1.46
	SD	0.21	0.27	0.41	0.34	0.15	0.35	0.18
Rg	mean	19.65	19.66	19.66	19.61	19.60	19.74	19.55
	SD	0.08	0.08	0.08	0.10	0.07	0.08	0.07
RMSF	mean	1.01	0.94	0.98	0.95	0.79	1.00	0.83
	SD	0.78	0.71	0.82	0.65	0.54	0.63	0.54

* The initial coordinate used a reference for RMSD calculation.

#### 3.2.2 Flexibility and fluctuations.

**Fig 7:**
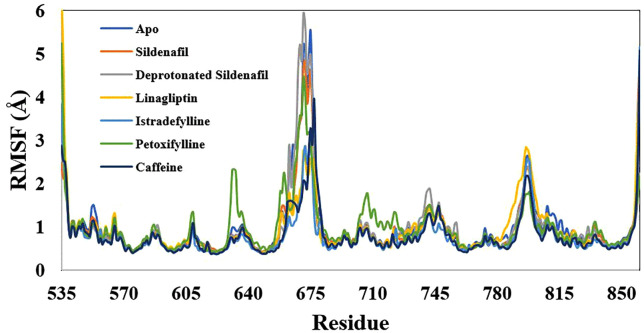
Root-mean-square fluctuation for the simulated xanthine derivatives-phosphodiesterase 5 complexes along the 300 ns trajectory.

**Fig 8:**
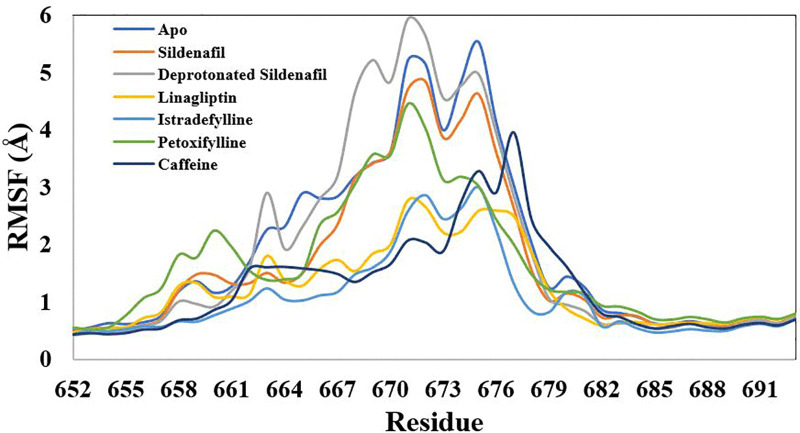
Embellished representation of the Root-mean-square fluctuation for the simulated xanthine derivatives-phosphodiesterase 5 complexes over the 100 ns trajectory.

The RMSF values revealed the significance of the residues of H-loop located between 660 and 686, demonstrating greater flexibility compared to the other regions of the enzyme, as illustrated in Fig 7. Several xanthine ligands under investigation demonstrated a significant reduction in the oscillations of this loop when compared to the apo state. Linagliptin, istradefylline, and Caffeine were effective, showing markedly lower RMSF peaks and thus inducing a more rigid and stable conformational change (Fig 8). The reference inhibitor, sildenafil, also reduced these fluctuations, but to a lesser extent than the xanthine derivatives. Whereas, the deprotonated sildenafil had the opposite effect, dramatically increasing the flexibility of this region beyond even that of the apo protein. This suggests that the deprotonated ligand induces the instability of the PDE-5, which consistent with unfavorable acceptor-acceptor interaction, as verified by molecular docking analysis.

#### 3.2.3 Hydrogen bonds analysis.

Hydrogen bond occupancy for xanthine derivative-phosphodiesterase 5 (PDE-5) complexes during 300 ns production runs is presented in Table 3. Among the PDE-5 active site residues, Gln817 demonstrated the highest tendency to form hydrogen bonds with all the investigated compounds. It played a dual role as both a hydrogen bond donor and acceptor. This was followed by the hydrogen bond donor Phe786 and the hydrogen bond acceptors Ile813, Val782, and Ser663. Ser663 also acted as a donor with linagliptin. Asn662 was highly significant in stabilizing the linagliptin ligand, showing a hydrogen bond occupancy of 42% throughout the simulation. Furthermore, istradefylline exhibited a higher hydrogen bond occupancy with Gln817, which was comparable to the hydrogen bond formed between Gln817 and the reference inhibitor, sildenafil.

**Table 3:** Hydrogen bond occupancy for xanthine derivative-phosphodiesterase 5 complexes during 300 ns production runs.

Acceptor	Donor	Occupancy (%)	Average Distance	Average Angle
Apo				
ILE_813@O	GLN_817@N	69	2.86	158
VAL_782@O	PHE_786@N	17	2.91	158
Sildenafil				
GLN_817@O	LIG_503@N	90	2.80	159
ILE_813@O	GLN_817@N	73	2.86	157
LIG_503@O	GLN_817@N	41	2.89	161
SER_663@O	ILE_665@N	19	2.87	146
VAL_782@O	PHE_786@N	15	2.92	157
Deprotonated sildenafil				
ILE_813@O	GLN_817@N	68	2.85	157
VAL_782@O	PHE_786@N	22	2.92	156
HID_613@N	ASN_661@N	18	2.93	153
LIG_503@N	GLN_817@N	11	2.90	152
LIG_503@O	GLN_817@N	8	2.85	157
LIG_503@O	GLN_817@N	5	2.87	51
Linagliptin				
ILE_813@O	GLN_817@N	66	2.86	157
LIG_503@O	ASN_662@N	42	2.88	161
VAL_782@O	PHE_786@N	27	2.90	156
ASN_662@O	SER_663@N	23	2.85	144
HID_613@N	ASN_661@N	13	2.91	154
Istradefylline				
LIG_503@O	GLN_817@N	74	2.85	161
ILE_813@O	GLN_817@N	73	2.85	157
VAL_782@O	PHE_786@N	23	2.92	158
SER_663@O	ILE_665@N	18	2.86	143
HID_613@N	ASN_661@N	10	2.94	157
LIG_503@O	TYR_612@OH	8	2.75	164
Petoxifylline				
ILE_813@O	GLN_817@N	67	2.87	159
VAL_782@O	PHE_786@N	22	2.91	154
SER_663@O	ILE_665@N	12	2.87	144
Caffeine				
ILE_813@O	GLN_817@N	76	2.85	157
VAL_782@O	PHE_786@N	25	2.91	159
SER_663@O	ILE_665@N	23	2.88	147
HID_613@N	ASN_661@N	18	2.93	157
LIG_503@O	LEU_725@N	7	2.92	162

### 3.3. The binding free energies and per-residue energy decomposition

Table 4 presents the binding free energies (ΔG) along with the individual energy components for the xanthine derivatives, sildenafil, and its deprotonated form bound to the PDE-5 receptor. To provide a comprehensive estimation of free binding energy, we employed both the MMGBSA and MMPBSA methods. For the majority of the PDE-5 complexes, the MMPBSA method—which is typically thought to be more accurate—predicted more advantageous binding energies than MMGBSA. The study identifies van der Waals interactions as the primary factor mechanisms behind the interactions, ranging from −25 to −59 kcal/mol. The electrostatic component (ΔE_elec_) is also favorable, but it is largely offset by unfavorable polar solvation energy (ΔE_sol_).

**Table 4:** The MMPBSA and MMGBSA terms for the binding energy for xanthine derivative- phosphodiesterase 5 complexes.

PDE-5 complex type	Calculation Method	E_vdw_	E_elec_	E_sol_	ΔH	TΔS	ΔG (kcal/mol)
Sildenafil	MMGBSA	−55.92	−17.89	24.65	−49.1	10.85	−38.3 ± 5.2
	MMPBSA	−55.92	−4.47	4.39	−56.01	6.97	−49.0 ± 3.1
Deprotonated Sildenafil	MMGBSA	−55.32	−47.35	67.11	−35.55	26.06	−9.5 ± 3.9
	MMPBSA	−55.32	−11.84	15.37	−51.78	12.02	−39.8 ± 3.0
Linagliptin	MMGBSA	−59.03	−46.56	65.92	−39.68	4.25	−35.4 ± 2.8
	MMPBSA	−59.03	−11.64	20.64	−50.03	1.89	−48.1 ± 2.4
Istradefylline	MMGBSA	−53.83	−14.42	33.33	−34.92	10.70	−24.2 ± 3.6
	MMPBSA	−53.83	−3.61	9.36	−48.08	3.66	−44.4 ± 2.9
Petoxifylline	MMGBSA	−28.67	−46.67	32.45	−42.89	9.68	−33.2 ± 3.6
	MMPBSA	−28.67	−11.67	9.55	−30.79	6.77	−24.0 ± 3.0
Caffeine	MMGBSA	−24.99	−42.64	34.85	−32.78	13.86	−18.9 ± 4.5
	MMPBSA	−24.99	−10.66	10.61	−25.04	9.08	−16.0 ± 3.4

Overall, the results reveal that linagliptin is the most potent inhibitor among the xanthine derivatives studied, with a binding energy comparable to that of the reference inhibitor sildenafil and surpassing its deprotonated form. The protonation state of sildenafil is crucial, as its deprotonated form accounts for 37.06% of the total molecules at a physiological pH of 7.4. Furthermore, istradefylline showed strong binding affinity, while pentoxifylline and caffeine were the slightly exhibited higher binding free energy compared to linagliptin and sildenafil (Table 3).

A per-residue binding free energy decomposition analysis was performed to identify the critical residues involved in ligand binding. The data presented in Table 5 and Table 6 illustrate distinct interactions for linagliptin with BDE-5 residues, which is aligned in parallel with the reference inhibitor sildenafil. The sildenafil exhibited a greater affinity for interaction with the residues Phe 820, Gln 818, Val 782, and PHE 786, while demonstrating a relatively lower affinity for the residues LEU 804, LEU 765, and LEU 725. Linagliptin demonstrated a favorable interaction with the residues ASP 764, ASN 661, and ASN 662, while exhibiting diminished affinity for the residues GLU 682, Tyr 664, Lys 770, and Ser 815. Furthermore, linagliptin was also shown to have unstable interactions with catalytic metals, specifically zinc and magnesium. These interactions are significant and may effectively block their accessibility.

**Table 5:** The per-residue contributions to the binding effective energy decomposition (kcal/mol) of PDE-5-sildenafil complex.

Residue	MMGBSA	MMPBSA
TYR 612	−0.9 ± 6.3	−1.1 ± 6.2
HID 613	−0.0 ± 6.2	−0.2 ± 6.0
ASN 661	−0.8 ± 7.7	−0.8 ± 6.9
ASN 662	0.2 ± 8.9	−0.3 ± 7.9
SER 663	−0.7 ± 6.3	−1.0 ± 5.8
ILE 665	−1.1 ± 6.5	−0.8 ± 6.3
ARG 667	−0.1 ± 20.7	0.1 ± 17.4
LEU 725	−1.1 ± 5.3	−0.8 ± 5.2
LEU 765	−1.4 ± 5.6	−1.5 ± 5.6
ALA 767	−0.1 ± 4.0	0.0 ± 4.0
ILE 768	−0.9 ± 5.5	−0.8 ± 5.5
GLN 775	−0.2 ± 6.0	0.3 ± 5.9
ILE 778	−0.4 ± 5.3	−0.4 ± 5.3
ALA 779	−0.6 ± 4.0	−1.0 ± 3.9
VAL 782	−2.6 ± 5.0	−2.4 ± 5.0
ALA 783	−0.4 ± 3.8	−0.4 ± 3.7
PHE 786	−1.9 ± 5.7	−2.5 ± 5.7
LEU 804	−1.7 ± 5.7	−1.8 ± 5.6
ILE 813	−0.8 ± 5.9	−0.8 ± 5.8
MET 817	−0.7 ± 5.2	−0.3 ± 5.1
GLN 818	−3.3 ± 5.6	−5.7 ± 5.5
PHE 820	−4.0 ± 5.3	−4.4 ± 5.3
Ligand	−25.4 ± 10.3	−32.0 ± 10.1

**Table 6:** The per-residue contributions to the binding effective energy decomposition (kcal/mol) of PDE-5-linagliptin complex.

Residue	MMGBSA	MMPBSA
TYR 612	−0.9 ± 6.8	−0.1 ± 6.6
HID 613	−0.3 ± 7.5	1.1 ± 7.4
HID 617	−0.6 ± 6.8	−0.7 ± 6.7
ASP 654	−5.1 ± 8.6	−13.1 ± 7.7
HIE 657	−0.6 ± 6.9	0.6 ± 6.7
ARG 658	0.1 ± 14.8	8.6 ± 11.8
VAL 660	−0.8 ± 5.2	−0.9 ± 4.9
ASN 661	−2.6 ± 5.7	−4.6 ± 5.3
ASN 662	−3.2 ± 5.7	−5.3 ± 5.5
SER 663	0.0 ± 4.6	0.2 ± 3.9
MET 681	−1.8 ± 3.9	−1.9 ± 3.7
GLU 682	−2.9 ± 9.9	−3.9 ± 9.3
HIE 684	0.1 ± 5.0	0.1 ± 4.6
HID 685	−1.6 ± 5.4	0.1 ± 5.4
THR 723	−0.6 ± 5.2	−2.4 ± 5.0
ASP 724	−0.8 ± 8.2	−9.9 ± 6.8
LEU 725	−2.3 ± 5.3	−1.5 ± 5.3
ASP 764	−2.8 ± 12.2	−18.4 ± 11.4
LEU 765	−0.7 ± 5.5	−0.9 ± 5.5
VAL 782	−1.1 ± 4.1	−1.0 ± 4.0
PHE 786	−1.7 ± 5.0	−1.2 ± 4.9
GLN 789	−0.2 ± 10.6	−0.1 ± 10.3
LEU 804	−0.2 ± 4.3	−0.4 ± 4.1
MET 805	−0.0 ± 5.2	−0.2 ± 5.1
PHE 820	−0.6 ± 5.9	−0.6 ± 5.9
ZN 501	7.1 ± 14.6	34.8 ± 11.6
MG 502	15.5 ± 14.7	6.1 ± 11.6
Ligand	−30.4 ± 13.5	−43.0 ± 13.2

### 3.4. Convergence and reproducibility

**Fig 9:**
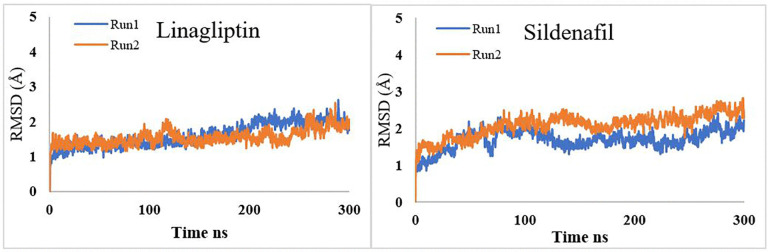
Root-mean-square deviation (RMSD) for the backbone carbon atoms of the replicate simulation of linagliptin and sildenafil-phosphodiesterase 5 complexes.

The reliability of our findings was confirmed using two independents, duplicate 300 ns MD simulations to the sildenafil and linagliptin complexes. Each replicate was initiated from the same minimized and equilibrated. The convergence and reproducibility of the simulations were assessed by comparing the RMSD data across the two trajectories for each system. Fig 9 shows RMSD for the backbone carbon atoms of the replicate simulations for both systems, based on the starting frame as a reference.

The overall results of the independent simulations were presented in Table 7. This strong agreement confirms that the MD protocol provides a reproducible description of the system’s dynamics, and that the simulations have robustly sampled the dominant conformational state of each complex.

**Table 7:** Post MD analysis of the replicates of the replicate simulation of linagliptin and sildenafil-phosphodiesterase 5 complexes during 300 ns production runs.

Analysis	Sildenafil (Run 1)	Sildenafil (Run 2)	Linagliptin (Run 1)	Linagliptin (Run 2)
RMSD*	1.7 ± 0.3	2.1 ± 0.3	1.6 ± 0.3	1.5 ± 0.2
RMSF	0.9 ± 0.7	1.0 ± 0.7	1.0 ± 0.7	0.9 ± 0.6
Rd	19.7 ± 0.1	19.5 ± 0.1	19.6 ± 0.1	19.7 ± 0.1
MMPBSA	−49.0 ± 3.1	−46.4 ± 3.0	−45.6 ± 4.3	−44.3 ± 5.2
MMGBSA	−38.3 ± 5.2	−32.2 ± 3.4	−23.6 ± 5.9	−13.6 ± 7.0

* The initial coordinate used a reference for RMSD calculation.

The primary challenge in identifying PDE-5 inhibitors is to ensure efficacy while minimizing side effects. Consequently, the prevailing attention among several researchers is focused on finding inhibitors derived from natural products [11,38]. Of these, nutraceutical lunamarine from watermelon [39], verbascoside and hesperidin [40], phenanthrene derivatives [41], total flavonoids of epimedium [42], citrulline derived from citrullus lanatus [43], and isoflavones and biflavones [44]. In addition to the synthetic compounds, exhibited PDE-5 inhibitory activity, such as monocyclic pyrimidinones [45], pyrazolopyrimidinone [46], thienopyrimidines [47], furyl/thineyl pyrroloquinolones based on natural alkaloid perlolyrine [48], diaminoquinazoline and N2, N6-diaminopurine scaffolds [49], pyrazolo [3, 4-d] pyrimidinone derivatives [50], thiazolopyrimidine derivatives [51], pyrazolopyrimidinone [52], multifunctional isosteric pyridine analogs-based 2-aminothiazole [53], pyridoindole derivatives [54], furoxan coupled spiro-isoquinolino piperidine derivatives [55]. In addition to synthetic compounds based on natural products, such as evodiamine derivatives [56]. Xanthine derivatives have a broad spectrum of bioactivity, encompassing anticancer, antibacterial, antiviral, and antileishmanial effects [19,20,57–62]. Interestingly, a synthesized compound-based xanthine scaffold has been studied as PDE-5 inhibitors [63].

The present study examines the potential of xanthine derivatives as a PDE-5 inhibitor. All xanthine derivatives exhibited a high tendency to form hydrogen bonds with the residues placed in the active range indicated by dynamic simulations of sildenafil and PDE-5 complex. However, some xanthine derivatives, such as istradefylline and pentoxifylline, do not lower the fluctuations of the active residues, even though they showed good binding scores with the PDE-5 receptor. These observations suggested that the xanthine small molecular weight could act as an agonist or a PDE-5 simulator. However, Linagliptin exhibits significant efficacy as a PDE-5 inhibitor, presenting a promising opportunity for the treatment of ED and pulmonary hypertension. Furthermore, the current literature signifies this finding, as indicated by the fact that the PDE5 inhibitors are effective against melanoma and lung cancer. It is also having anti-diabetic, anti-inflammatory, antioxidant, and immunomodulatory effects as well as benefits for Alzheimer’s disease [64–70]. Linagliptin is a dipeptidyl peptidase 4 inhibitor used in the treatment of type 2 diabetes, recognized for its safety profile. Recent studies suggest that it is advantageous to enhance cardiac function [67] and inhibit human fibroblast activation protein [71]. Previous in vivo investigations have also demonstrated its anti-inflammatory and antioxidative effects.

## Conclusion

To sum up, the significance of xanthine derivatives as phosphodiesterase 5 (PDE-5) inhibitors was evaluated using different computational methods, including molecular docking, molecular dynamics, binding free energy, and per-residue energy decomposition. The comprehensive findings indicate that linagliptin stands out as the most effective inhibitor among the xanthine derivatives assessed as PDE-5 inhibitors. It reduces the flexibility of the important H-loop in PDE-5 and binds more closely to the catalytic zinc and magnesium metal ions. In addition, linagliptin was shown to be a better stabilizing agent of PDE-5 with one predominant protonated state, unlike sildenafil, which showed an unstable deprotonated state due to unfavorable acceptor-acceptor interactions. Thus, it is recommended that additional research be conducted on the potential repurposing of linagliptin as a PDE-5 inhibitor to alleviate the identified adverse effects linked to current PDE-5 inhibitors [72–74].

## Supporting information

S1 Data Raw data for Figs 5–8 and Table 2. (XLSX)

S2 Data Raw data for Fig 9 and Table 7. (ODS)
